# The Truth about Traumatic Hernia*Read before the Section of Surgery, State Medical Association of Texas, San Antonio, May 15, 1918.

**Published:** 1918-09

**Authors:** Bacon Saunders

**Affiliations:** Fort Worth, Texas


### The Truth About "Traumatic" Hernia.*

BY BACON SAUNDERS, M. D., F. A. C. S., FORT WORTH, TEXAS.

---

*Read before the Section of Surgery, State Medical Association of Texas, San Antonio, May 15, 1918.

It is not the present purpose to discuss any method of treatment, either so-called conservative, or operative anl radical, or to mention even briefly the questions of diagnosis and prognosis, it being taken for granted that surgical opinion is a very decided and definite unit regarding treatment; that diagnosis is a matter of individual skill; and that prognosis must be founded on the sum total of competent professional experience.

---

*Read before the Section of Surgery, State Medical Association of Texas, San Antonio, May 15, 1918.

From the time of Hippocrates and Galen down the ages of surgical history, hernia has been the subject of perennial interest. Pages, chapters and volumes have been written until the literature on the subject forms no small part of every surgical library. Indeed, such writings are not confined to surgical texts, but occupy considerable space in all standard works on anatomy as well. A study of the subject as presented in both surgical and anatomical texts gives abundant reason for the conclusion that man's physical condition has been closely observed and recorded and that he has been the victim of hernia since a time when the knowledge of man "runneth not to the contrary." A striking thing in the history of hernia and that furnishes food for careful thought, is that anatomists from the days of Vesalius on down describe it as a natural anatomical condition, else why should they have anything at all to say about it, since anatomy deals with natural, not pathologic, or diseased structures. To borrow the well-known advertising slogan, there must be a "reason." This reason must account for the fact that at least one person in every twenty of the human family has a hernia at some time in life, and that eight-tenths of the subjects are of the masculine gender. There must also be "a reason" for this wonderful disproportion between males and females as to being the subjects of hernia. It can only be explained by finding in the male some natural anatomical condition favoring the development of hernia that practically does not exist in the female. To put the same thought in another way, when women do have hernia, where do they have it, and why? Answers to these questions go a long way toward explaining the whole modus operandi of the development of hernia.

Because hernia is so common and for the further reason that there is such a general misconception on the part of both the profession and the public as to the true factors in the causation of practically every case of hernia, and particularly so-called "traumatic" hernia, and in consideration of the immense economic importance of the whole subject, it was thought worth while to give it a brief but carefully considered presentation. In order to do this understandingly, it is necessary to call to mind a few well-known but easily forgotten facts in the anatomy of human development in the early months.

Some time prior to the middle intrauterine life, the testicle begins gradually to descend from its position high up in mid-abdomen, one on either side of the spinal column. In the third month it is on the false pelvis; at the sixth month it is in contact with the abdominal wall near the internal inguinal ring; at the ninth it is usually well in the scrotum. Its descent is probably caused by body development, it being drawn by the gubernaculum testis obliquely downward between the layers of the abdominal wall, into the peritoneal pouch formed along the course of the gubernaculum, to the scrotum. Inside the glove-finger-like pouch of peritoneum, trailing behind the testicle, are the vas deferens, blood vessels, nerves and lymphatics, which together form the spermatic cord, the diameter of which is about one-sixteenth that of the corresponding testicle. It is not material to the conditions leading to the future development of hernia whether the testicle made its way through as pre-existing pouch, the vaginal process, as the embryologists tell us is the case, or pushed the pouch before it, the fact remains that it made the journey and the way was thus opened up as late as the sixth to ninth month of gestation.

In the female there is a descent of no similar organ from inside to outside the abdomen, and leaving behind this tell-tale peritoneal prolongation called in the male the infundibular process. The nearest approach to it in the female is a somewhat similar process of peritoneum carried by the femoral vessels underneath Poupart's ligament into the space known as the crural canal to emerge at another potential point called the femoral ring. This makes the perfectly natural anatomical reason why when a woman has a hernia, eight times out of ten it is femoral rather than inguinal. The much less frequent cases of hernia in the canal of Nuck develop similarly in the peritoneal sheath that envelops the round ligament. These facts in the anatomical development of the fetus must be constantly borne in mind in order to get an accurate conception of the mechanism in the development of any case of hernia.

Going back to the journey of the testicle through the abdominal wall down to the scrotum, the split in the muscles and fascia where it enters the abdominal wall, after leaving the cavity is known as the internal ring; the space through which it passes between the muscles, the inguinal canal; where it emerges at the weakest point in the wall underneath the conjoined tendon, the external ring. We are wont to think of these as actual rings and the canal as a bona fide canal, when as a matter of fact, the rings, as nature left them, are only pseudo-rings and the canal a canal in reality only when occupied by a foreign body. The canal is, therefore, only a potential canal and the rings are non-resistant spaces or slits in the abdimonal wall left after separation of the fascial and muscular layers by the descending testicle. While the descending testicle may be said to figuratively bore its way downward between and through the muscles and fascia of the abdominal wall, preceded by the peritoneum, the latter structure being exteremely elastic at that period of embryonic life, it must be borne in mind that the whole process is a developmental one and not to be compared to any imaginary similar occurrence taking place in post-uterine life. The infundibular process of peritoneum in the individual of ideal physical development begins to contract and adhere in the latter third of uterine life and by birth is closed from internal ring to testicle, except the part one-sixteenth its original caliber occupied by the cord. This process takes place in various degrees of completeness according to the approach of the individual to a perfect standard of anatomic development, ranging all the way between the open, patulous pouch from internal ring to testicle, and the slight dimple or depression at the seat of the internal ring. The condition is a perfectly natural one, just as much so as the shape of the nose, or the length of the femur, and renders every male person, except those who are anatomically perfect, and who never develop it, predisposed by nature to inguinal hernia. We are anatomically predisposed to hernia, just as for a similar perfectly natural cause we are physiologically predisposed to measles, whooping cough and chicken pox. The anatomically perfect, it may be said, then, in all truth and fairness, are the only immunes to hernia. Unfortunately for the human race, a large number have not been blessed with the ideally perfect development. It is this less fortunate class who are thus liable to develop hernia "in the natural course of human events," any time in their lives.

It has already been said that in the perfectly developed specimen of physical manhood the rings are only slits and the canal a potential space that is not in reality a canal except when occupied by a foreign body. In such instances of perfect development both the pseudo-rings and the potential canal are kept permanently and effectively closed by intra-abdominal pressure. The case of the individual whose development is not so ideal is quite another story. In one instance the process may be so imperfect that the person is born with a hernia. In another it appears without provocation when the child is a month or three months or twelve months, or the youth is five, ten or fifteen years old. Will the most enthusiastic advocate of "traumatic" hernia claim an injury or traumatism to these infants and children under such circumstances? If so, he either does not or will not know anything about the mechanism of hernia. "Ephraim is joined to his idols, let him alone." The hernia in such instances appears as a perfectly natural result of the ordinary processes of life and is of a character predetermined by the want of perfect anatomical structure in the individual case. The only sure way such persons can avoid hernia of some degree, developing at some time in their lives, is to die before it shows up.

Every hernia must have two things before it can exist—a sac and the viscus or structure in the sac constituting the contents; no sac, no contents, no hernia. The sac of every hernia is made by the gradual redilating of the partially closed original infundibular process of peritoneum in the indirect form or by the same process of slow dilation of the peritoneal lining of the depression in the abdominal wall behind the weak place known as Hesselback's triangle. It is well known that the peritoneum which was elastic and pliant in early uterine life becomes inelastic and resistant after birth. It is, therefore, inconceivable that the sac of a hernia can be formed by one violent act driving the abdominal organs against the peritoneum without tearing it wide open. If such an untoward event were to occur there would not be a hernia with its essential and omnipresent peritoneal sac, but a protrusion of the abdominal viscus through the lacerated wall uncovered by peritoneum, a denoument which few, if any of us, have seen or will ever see. Such a condition would be a "rupture" indeed and would be followed by signs and symptoms both local and general that would be unmistakable. Instead of such an unfortunate catastrophe, hernia comes about as the result of the slow stretching of the peritoneum at its weak and unsupported parts already described, caused by the many times repeated wedge-like pressure of the abdominal contents. This gentle distending process must go on a long time before the hernial sac can be developd. This wedge-like pressure of an intra-abdominal organ in the ordinary functions of life causes many excursions of such organ into and out of the gradual forming sac, so persistently and painlessly that the subject makes no discrimination between them and perfectly natural and normal physical processes, with no thought that he is the victim of a developing hernia until some day a sudden cough, or sneeze, or strain at stool, or an emptying of the bladder, or even, perchance a jump or run, or some other unusual exertion causes the contents to enter the sac suddenly, thus surprising the muscles into contraction. The contraction causes slight pain, which, in turn calls the subject's attention to the locality and he discovers for the first time a hernia that has been really developing for weeks, months or even years, without his notice. Nothing has been torn, bursted or ruptured.

In this view of the mechanism and correct development of hernia, it is very unfortunate, in the interest of scientific truth, that the word "rupture" should ever have been used in connection with it, since the word gives rise to an idea that is entirely at variance with the facts and the true steps of its formation. The word describes a condition that does not exist, in fact, never took place, and is, therefore, erroneous, untrue and misleading and ought to be eliminated from all descriptive texts and discussions of the subject.

That many cases of hernia do not develop or "show up" until middle, active life, is a perfectly natural result of the method of their development, and this is the class of cases about which the camouflage of "traumatic" hernia has appeared. The ignorance of the people as to the real cause of hernia and the perfectly natural dread of their "insides coming out"; sometimes, candor compels me to say, an unholy desire for gain, coupled with the careless and indiscriminate, not to say, ignorant, use of the word "rupture" by the majority of doctors, is the perfectly apparent cause of practically ever case of alleged "traumatic" hernia. It is not argued for a moment, as will be perceived from statements made above, that every individual claiming such a condition is aware that the claim is unjust. Many of them really believe they have been "ruptured" because of the mistaken idea that hernia is always caused that way, and besides, more often than otherwise, "the doctor said so."

People are not supposed to know anatomy or understand the mechanism of the production of hernia, but doctors are in honor bound to do both. They should be equally honor-bound to educate the public to a reasonable understanding of the true conditions operating in the development of every case of hernia. They should know and be able to show to their clients the anatomical and physical impossibility of a hernia being the result of a single primary injury of any kind no matter how severe.

It can, therefore, be said with confidence from this understanding of the subject that hernia is not due to traumatism or injury, but is a perfectly natural occurence that may develop in any individual not made proof against it, or immune to it, by a perfect anatomical organism. Justice, fair play and simple, unvarnished truth all demand that doctors, especially, cease the senseless prattle about this thing they call "rupture" and "traumatic" hernia.

